# A Tara Gum/Olive Mill Wastewaters Phytochemicals Conjugate as a New Ingredient for the Formulation of an Antioxidant-Enriched Pudding

**DOI:** 10.3390/foods11020158

**Published:** 2022-01-08

**Authors:** Umile Gianfranco Spizzirri, Paolino Caputo, Cesare Oliviero Rossi, Pasquale Crupi, Marilena Muraglia, Vittoria Rago, Rocco Malivindi, Maria Lisa Clodoveo, Donatella Restuccia, Francesca Aiello

**Affiliations:** 1 Dipartimento di Farmacia e Scienze della Salute e della Nutrizione, Dipartimento di Eccellenza 2018–2022, Università della Calabria, Ed. Polifunzionale, 87036 Rende, Italy; g.spizzirri@unical.it (U.G.S.); vittoria.rago@unical.it (V.R.); rocco.malivindi@unical.it (R.M.); francesca.aiello@unical.it (F.A.); 2 Dipartimento di Chimica e Tecnologie Chimiche, Università della Calabria & UdR INSTM della Calabria, 87036 Rende, Italy; paolino.caputo@unical.it (P.C.); cesare.oliviero@unical.it (C.O.R.); 3 Dipartimento Interdisciplinare di Medicina, Università degli Studi Aldo Moro Bari, Piazza Giulio Cesare 11, 70124 Bari, Italy; pasquale.crupi@crea.gov.it (P.C.); marialisa.clodoveo@uniba.it (M.L.C.); 4 Dipartimento di Farmacia-Scienze del Farmaco Università degli Studi di Bari, Campus Universitario E. Quagliarello Via Orabona 4, 70125 Bari, Italy; marilena.muraglia@uniba.it

**Keywords:** olive mill wastewater, polyphenols, antioxidant features, tara gum, pudding, rheological properties

## Abstract

Olive mill wastewater, a high polyphenols agro-food by-product, was successfully exploited in an eco-friendly radical process to synthesize an antioxidant macromolecule, usefully engaged as a functional ingredient to prepare functional puddings. The chemical composition of lyophilized olive mill wastewaters (LOMW) was investigated by HPLC-MS/MS and ^1^H-NMR analyses, while antioxidant profile was in vitro evaluated by colorimetric assays. Oleuropein aglycone (5.8 μg mL^−1^) appeared as the main compound, although relevant amounts of an isomer of the 3-hydroxytyrosol glucoside (4.3 μg mL^−1^) and quinic acid (4.1 μg mL^−1^) were also detected. LOMW was able to greatly inhibit ABTS radical (IC_50_ equal to 0.019 mg mL^−1^), displaying, in the aqueous medium, an increase in its scavenger properties by almost one order of magnitude compared to the organic one. LOMW reactive species and tara gum chains were involved in an eco-friendly grafting reaction to synthesize a polymeric conjugate that was characterized by spectroscopic, calorimetric and toxicity studies. *In vitro* acute oral toxicity was tested against 3T3 fibroblasts and Caco-2 cells, confirming that the polymers do not have any effect on cell viability at the dietary use concentrations. Antioxidant properties of the polymeric conjugate were also evaluated, suggesting its employment as a thickening agent, in the preparation of pear puree-based pudding. High performance of consistency and relevant antioxidants features over time (28 days) were detected in the milk-based foodstuff, in comparison with its non-functional counterparts, confirming LOWM as an attractive source to achieve high performing functional foods.

## 1. Introduction

In recent years, to increase beneficial effects on the human health of many foodstuffs and beverages, some health-promoting natural substances with antioxidant activity were used as high- performing functional ingredients [1]. In particular, the exploitation of agro-industrial wastes represents a smart opportunity for sustainable growth and the scientific research so far has made considerable efforts to reuse and enhance agro-food waste as a source of bioactive compounds for large use in cosmetics, pharmaceuticals and in the agri-food field [2,3]. Our scientific experience suggests that antioxidant features of biomolecules in the olive mill wastewaters (OMW), as well as leaves, pomace and pits discharged from the EVOO production process, are partially recycled and usefully employed in the nutraceutical and pharmaceutical fields [4,5]. To this regard, literature data highlighted that the employment of macromolecular antioxidants displayed some undoubted advantages such as increased stability and slower degradation rate, compared to the compounds with low molecular weight [6]. In particular, polysaccharides are involved in the preparation of different foodstuffs specifically in a wide variety of milk-based desserts such as pudding due to their ability to influence the rheological and texture characteristics of the final product [7]. Puddings are semisolid dairy desserts that are generally milk protein-based starch pastes [8]. Nowadays, on the market are available ready-to-eat puddings or alternatively instant pudding powders able to get a gel structure by dissolving, in a relatively short period time, in cold water or milk and a subsequent cooling step [9]. The formulation of the commercial pudding powders usually consists of hydrocolloids, starch, flavors, sugars and colorants [10]. 

In this article, the possibility of using the polyphenolic components present in the OMW to obtain a functional polymer able to be employed in the preparation of puddings which display a high performance of consistency (mechanical properties) and relevant antioxidant features over time was investigated. For this purpose, tara gum (TG) was functionalized with reactive components of the OMW allowing the synthesis of a polymeric conjugate, representing a basic component of the pudding formulation. TG was picked up from the tara seed endosperm and based on (1 → 4)-β-mannose chain with an unit of (1 → 6)-α-galactose (mannose to galactose ratio equal to 3:1) [11]. In conjunction with the starch, TG allows the gelling process of the food hydrocolloid [12]. TG has been largely proposed in the food field as encapsulating material of bioactive molecules by freeze-drying and spray-drying methods [13] or in the preparation of crosslinked polymeric carriers able to deliver nutraceuticals [14]. Our challenge was to synthesize a TG-based macromolecular antioxidant by a molecular grafting reaction using a redox couple (H_2_O_2_/ascorbic acid) as an initiator system and involving the heteroatoms in the polymer chains of the polysaccharide and the polyphenol moieties of the active compounds in the OMW. 

The polymer conjugate was used as a thickening agent in the preparation of pear puree-based pudding, which has been characterized over time by rheological tests and whose antioxidant profile has also been depicted. The employment of the antioxidant TG by increasing the total content of phenolic groups in the final product and preserving the biologically active molecules derived from fruit should guarantee a food product with a greater antioxidant capacity, practically unchanged over time. Finally, rheological analyses in asymptotic kinematics made it possible to evaluate the effects of the functionalized polysaccharide on the colloidal structure of the pudding. Specifically, the weak gel model was applied to analyze the strength of the gel and its coordination in quantitative terms. 

## 2. Materials and Methods

### 2.1. Chemicals 

Tara gum, pectin esterified from citrus fruits with a degree of methoxylation of 55–70%, gallic acid, (+)-hydrated catechin, L-ascorbic acid, hydrogen peroxide (H_2_O_2_, 30% *v*/*v*), Folin–Ciocalteu reagent, carbonate sodium (Na_2_CO_3_) radical 2,2′-diphenyl -1-picrylyhydrazyl (DPPH), radical 2,2′-azino-bis (3-ethylbenzothiazolin-6-sulfonic) (ABTS), potassium persulfate (K_2_S_2_O_8_), ammonium molybdate tetrahydrate ((NH_2_)_2_MoO_4_), sodium molybdate (Na_2_MoO_4_), sodium nitrite (NaNO_2_), sodium phosphate (Na_3_PO_4_), aluminum chloride (AlCl_3_), hydrochloric acid (HCl), sodium hydroxide (NaOH), absolute ethanol, methanol, Whatman No. 3 filter paper, dialysis membrane (MWCO: 12,000–14,000 Da), were purchased from Sigma Aldrich (Sigma Chemical Co., St. Louis, MO, USA). HPLC grade water, formic acid and acetonitrile were purchased from VWR (Chromasolv, VWR International Srl, Milano, Italy).

### 2.2. Sample Collection and Preparation

Green olives of Roggianella cultivar were harvested (October 2019) in the Northern of Calabria and processed on-site the next day (Oil mill Vinciprova srl in San Vincenzo la Costa (Cosenza, Italy)) using a semi-continuous Enorossi 150 traditional olive oil pressing system (Enoagricola Rossi, Calzolaro di Umbertide, Perugia, Italy) standardized to press a maximum of 150 kg of olives at a time. Olive mill wastewater (OMW) sample was collected and stored in 1.0 L low density polypropylene airtight containers at −18 °C until use. OMW were filtered and, subsequently, the filtrate was centrifugated (10 min at 10,000 rpm). Finally, the solution was frozen and dried by freeze-drying providing a dark colored vaporous solid (LOMW).

### 2.3. Chemical Characterization of Lyophilized Olive Mill Wastewaters

#### 2.3.1. HPLC-MS Analysis of Lyophilized Olive Mill Wastewaters 

HPLC 1100 system composed of a degasser, quaternary pump solvent delivery, thermostatic column compartment, auto-sampler, single wavelength UV-Vis detector and MSD triple quadrupole QQQ 6430 in a series configuration (Agilent Technologies, Palo Alto, CA, USA) was employed for the polyphenols analysis. LOMW was resuspended in 2 mL ethanol/water (1:1, *v*/*v*) to a final concentration of ~1.2 mg mL^−1^ and filtered through 0.2 μm pore size regenerated cellulose filters (VWR International Srl, Milano, Italy) and injected onto a reversed stationary phase column, Luna C_18_ (150 × 2 mm i.d., particle size 3 μm, Phenomenex, Torrance, CA, USA) protected by a C_18_ Guard Cartridge (4.0 × 2.0 mm i.d., Phenomenex). HPLC separation was carried out through a binary gradient consisting in (solvent A) H_2_O/formic acid 0.1% (*v*/*v*) and (solvent B) acetonitrile: 0 min, 10% B; 1 min, 10% B; 15 min, 30% B; 22 min, 50% B; 28 min, 100% B; 34 min, 100% B; 36 min, 10% B, followed by washing and re-equilibrating the column (with ~20 column volume). The column temperature was controlled at 20 °C and the flow was maintained at 0.4 mL min^−1^. UV-Vis detection wavelength was set at 280 nm.

Because polyphenols contain one or more hydroxyl and/or carboxylic acid groups, MS data were acquired in negative ionization mode with capillary voltage at 4000 V, using nitrogen as drying (T = 350 °C; flow rate = 9.0 L min^−1^) and nebulizing gas (40 psi). MS and MS/MS spectra were acquired in the range between *m*/*z* 50 and 1200. All data were processed using Mass Hunter Workstation software (version B.01.04; Agilent Technologies). UV absorption, retention times (RT), elution order and mass spectra (MS and MS/MS) were compared with those from pure standards (3-hydroxytyrosol, caffeic acid and *p*-coumaric acid) and/or matched with those already reported in the literature [15,16,17]. Then, the main revealed compounds were quantified by multiple reaction monitoring (MRM) as 3-hydroxytyrosol (R^2^ = 0.99923). Mass Hunter Optimizer software (version B.03.01; Agilent Technologies) (Table S1).

#### 2.3.2. H-NMR Analysis of Lyophilized Olive Mill Wastewaters 

The sample LOMW (16.7 mg) was dissolved in 0.6 μL of D_2_O (99.9% D). ^1^H-NMR spectrum was performed at 25 °C using a Brucker Advance 200 spectrometer of 300 MHz equipped with ^13^C/^1^H dual probe. The NMR experiments were recorded with a spectral width of 6983.240 Hz, an acquisition time of 10.20 s, a number of 64 scans, a relaxation time of 2 s and a pulse width of 7 s. The spectra were processed by XWIN-NMR.

#### 2.3.3. Polyphenols Total Content

Folin–Cicocalteu method was employed to evaluate available phenolic groups (APG) [18]. Different concentrations of an aqueous solutions of LOMW (6.0 mL) were added to 1.0 mL of Folin–Ciocalteu reagent and after 3 min, 3.0 mL of Na_2_CO_3_ (2.0 % *w*/*v*) was also added keeping the solution under stirring in the dark (time = 2 h) and spectrophotometrically measuring at 760 nm. APG was expressed as milligrams of catechin (CT) per gram of LOMW (mg CT/g LOMW), by using an equation obtained from a calibration curve of CT at different concentrations (8.0, 16.0, 24.0, 32.0 and 40.0 μM). The method of least square was used to calculate a calibration curve. Each measure was performed in triplicate and data expressed as means (±SD). UV-Vis absorption spectra were recorded with a Jasco V-530 UV/Vis spectrometer (Jasco, Tokyo, Japan).

#### 2.3.4. Phenolic Acid Content

Arnov test, with some modifications, was used to evaluate the phenolic acids content (PAC), expressed in milligrams of CT per gram of LOMW (mg CT/g LOMW) [19]. In a volumetric flask (10.0 mL) 1.0 mL of an aqueous solution of LOMW, 1.0 mL of HCl (0.5 mol L^−1^), 1.0 mL of Arnov’s reagent (sodium molybdate and sodium nitrite 0.1 mg mL^−1^), 1.0 mL of NaOH (1.0 mol L^−1^) and purified water were mixed. The absorbance of the solutions was measured by a spectrophotometer at 490 nm. Each measurement was performed in triplicate and data expressed as means (±SD).

#### 2.3.5. Flavonoid Content

Flavonoid content (FC) in the samples was determined by a spectrophotometric method reported in literature with some modifications [20]. In a volumetric flask (5.0 mL), 0.5 mL of LOMW and 0.150 mL of NaNO_2_ (5.0 % *w*/*v*) solution were mixed. After 6 min, 0.300 mL of a 6.0 % (*w*/*v*) AlCl_3_ was added and after 5 min, 1.0 mL of NaOH (1.0 mol L^−1^) was also added to the mixture by immediately measuring the absorbance (510 nm) against a control solution. FC in LOMW was expressed as milligrams of CT per gram of sample, by using a calibration curve of CT (Standard solutions concentration of CT equal to 10.0, 25.0, 50.0, 75.0, 100.0 μM). Each measurement was performed in triplicate and data expressed as means (±SD).

#### 2.3.6. Anthocyanin Content 

The anthocyanin content (AC) was determined by using the procedure reported in literature, with some modifications [21]. 1.0 g of LOMW was suspended in 10.0 mL of methanolic solution of HCl (1.0% *v*/*v*). The solution was incubated at 60 °C under stirring for 1 h and then filtered in a volumetric flask (10.0 mL). The absorbance was spectrophotometrically measured at 657 and 530 nm. The net absorbance was calculated according to the following equation:

Net absorbance = Absorbance at 530 nm − 0.25 (Absorbance at 657 nm)(1)

AC was calculated on the basis of cyanidin-3-glycoside (molecular weight equal to 449.1 g mol^−1^ and extinction coefficient of 29,600) [22]. AC was expressed in milligrams of anthocyanins per gram of LOMW (mg AC/g LOMW), according to the following equation:

AC (mg/g) = ((Net absorbance)/29,600) × MW × FT × (V/(Sample weight))(2)

where MW is the molecular weight of cyanidin-3-glycoside, FT is the dilution factor, V is the total volume (mL). Each measurement was performed in triplicate and data expressed as means (±SD).

#### 2.3.7. Antioxidant Properties

The antioxidant properties of LOMW were evaluated by using specific tests (inhibition capacity towards the lipophilic (DPPH) and hydrophilic (ABTS) radicals and to measure the total antioxidant activity (TCA)).

The scavenging activity of LOMW in an organic environment was evaluated in terms of decrease of the radical 2,2-diphenyl-1-picrylhydrazyl (DPPH^•^) concentration [23]. A total of 1.0 mL of hydro-alcoholic solutions (50:50 *v*/*v*) of LOMW were added to 4.0 mL of hydro-alcoholic mixture (50:50 *v*/*v*) and 5.0 mL of DPPH^•^ ethanolic solution (200 µM). The mixture was kept at 25 °C for 30 min and the residual concentration of the DPPH^•^ radical was spectrophotometrically evaluated at 517 nm. The percentage of inhibition of the DPPH^•^ radical was calculated according to the following equation:

Inhibition (%) = (A_0_ − A_1_)/A_0_ × 100 (3)

where A_0_ is the absorbance of the control solution prepared under the same conditions but without sample, while A_1_ is the absorbance recorded analyzing LOMW sample. The scavenging activity of LOMW against the lipophilic radical DPPH was expressed in terms of IC_50_. Each measure was performed in triplicate and data expressed as means (±SD).

The scavenging activity in the aqueous environment of LOMW was determined in terms of reduction of the radical 2,2′-azino-bis(3-ethylbenzothiazolin-6-sulphonic) (ABTS^•^) [24]. Aqueous solutions of LOMW were prepared, at different concentrations and 2.0 mL of the aqueous solution of the ABTS radical was added to 500 µL of each. The solutions were then kept in the dark for 6 min and the residual concentration of the ABTS radical was spectrophotometrically evaluated at 734 nm. The percentage of inhibition of the ABTS^•^ was calculated according to the Equation (3), while LOMW scavenging activity was expressed in terms of IC_50_. Each measure was performed in triplicate and data expressed as means (±SD).

Total antioxidant capacity (TAC) of LOMW was evaluated by mixing 300 µL of LOMW hydro-alcoholic solutions (50:50 *v*/*v*) with 1.2 mL of the reagent solution (28.0 mmol L^−1^ Na_3_PO_4_, 4.0 mmol L^−1^ (NH_4_)_2_MoO_4_, 0.6 mol L^−1^ H_2_SO_4_) [25]. The solutions were kept at 95 °C in the dark for 150 min and then by measuring the adsorbance at 695 nm. A calibration curve was constructed by the method of least square, using 8.0, 16.0, 24.0, 32.0 and 40.0 µM hydro-alcoholic solutions (50:50 *v*/*v*) of CT. The TAC was expressed in milligrams of CT per gram of LOMW (mg CT/g LOMW). Each measure was performed in triplicate and data expressed as means (±SD).

### 2.4. Synthesis of the Tara Gum Conjugate by Grafting Procedure

The synthesis of polymeric conjugate was carried out according to the methods reported in literature, with some modifications [26]. In a 100 mL glass flask, 0.250 g of tara gum was dissolved in 37.5 mL of purified water, then 12.5 mL of H_2_O_2_ (120 vol) and 0.3 g of ascorbic acid were added at 25 °C. After 2 h, LOMW (equivalent to 0.035 g of CT) was dissolved into the reaction flask. After 24 h, the mixture was introduced into dialysis tubes (MWCO: 12,000–14,000 Dalton) and dipped into a glass vessel containing distilled water for 48 h. The conjugate (PLOMW) was checked to be free of unreacted antioxidant and any other compounds by LC analysis after purification step. LC analysis was performed on a Knauer (Asi Advanced Scientific Instruments, Berlin, Germany) system equipped with two pumps Smartiline Pump 1000, a Rheodyne injection valve (20 μL) and a photodiode array detector equipped with a semi-microcell. The resulting solution was frozen and dried with ‘‘freezing-drying apparatus” to provide a vaporous solid. A control polymer, blank tara gum (BTG), was also prepared under the same conditions but without LOMW.

### 2.5. Characterization of Tara Gum Conjugate

#### 2.5.1. H-NMR Analysis of Tara Gum Conjugate 

The samples of PLOMW and BTG (5.8 mg) were dissolved in 0.6 μL of D_2_O (99.9% D). ^1^H-NMR spectra were performed at 25 °C using a Brucker Advance 200 spectrometer of 300 MHz equipped with a ^13^C/^1^H dual probe. The NMR experiments were recorded with a spectral width of 6983.240 Hz, an acquisition time of 10.20 s, a number of 64 scans, a relaxation time of 2 s and, a pulse width of 7 s. The spectra were processed by XWIN-NMR.

#### 2.5.2. Differential Scansion Calorimetry (DSC) 

The DSC studies were performed using a SETARAM 131 instrument. The amount of each sample was around 3–10 mg. Analyses were performed from 25 to 650 °C at a temperature scan rate of 10 °C min^−1^, under nitrogen flux.

The method used is set out in more detail below:

1. Isotherm at 25 °C for 20 min;
2. Heating from 25 to 650 °C at 10 °C min^−1^.

#### 2.5.3. Antioxidant Properties of the Tara Gum Conjugate

PLOMW conjugate was characterized in terms of available phenolic groups, phenolic acids and flavonoids content by the methodologies previously described, expressing the results as mg of CT per gram of PLOMW. Similarly, antioxidant performances were recorded by evaluation of TAC (expressed as mg of CT per gram of PLOMW) and scavenging activity if both aqueous (against ABTS radical) and organic (against DPPH radical) environments. 

#### 2.5.4. Toxicity of the Tara Gum Conjugate

##### Cell Culture

Balb/c 3T3 clone A31 and Caco-2 were purchased from ATCC, Manassas, VA, USA. Balb/c 3T3 clone A31 cells were maintained in DMEM with 10% Calf Bovine Serum (CBS; ATCC, Manassas, VA, USA) and 1% penicillin-streptomycin (10,000 unit/mL) at 37 °C in a 5% CO_2_ atmosphere. Caco-2 cells were cultured in DMEM with FBS 10% (*w*/*w*), non-essential amino acids (1% *w*/*w*), L-glutamine (1% *w*/*w*) and penicillin–streptomycin (1% *w*/*w*). The cells were grown in a humidified atmosphere (5% CO_2_) at 37 °C until 80% confluence and sub-cultured twice a week [27].

##### Neutral Red Uptake (NRU)

The *in vitro* 3T3 NRU test was performed as described by the ISO 10993-5:2009 “Biological evaluation of medical devices—Part 5: Tests for *in vitro* cytotoxicity”. Briefly, 2.5 × 10^4^ 3T3 cells/well were incubated in 96-well plates and cultured for 24 h at 37 °C and 5% CO_2_ in humidified hair with increases doses of BTG and PLOMW (0.39, 0.78, 1.56, 3.12, 6.25, 12.5 and 25 mg mL^−1^) overnight in DMEM. Cell viability was measured by neutral red uptake (NRU) assay [28]. The NRU assay provided an incubation (3-h) with neutral red (50 μg/mL in DMEM) followed by an extraction with acetic acid, ethanol and water (1:50:49 *v*/*v*/*v*). The absorbance was measured at 540 nm in a microplate reader Epoch (BioTek, Winooski, VT, USA). A percentage of viability was calculated as follows:

(4) $$\%\mathrm{Viability}=\frac{\left({\mathrm{Absorbance}}_{540\mathrm{nm}}\mathrm{test}\;\mathrm{material}\right)\;-\;{\mathrm{Absorbance}}_{540\mathrm{nm}}\mathrm{blank})}{\left({\mathrm{Absorbance}}_{540\mathrm{nm}}\mathrm{control}\right)\;-\;{\mathrm{Absorbance}}_{540\mathrm{nm}}\mathrm{blank})}$$

##### Cell Viability Assay

MTT staining assay was used to evaluate cell viability as described by Mosmann [29]. Briefly, 1 × 10^4^ Caco-2 cells/well were incubated in 96-well plates and cultured for 24 h at 37 °C and 5% CO_2_ in humidified hair with increases doses of BTG and PLOMW (3.12, 6.25, 12.5 and 25 mg mL^−1^); then, fresh MTT was added to each well and after 2 h of incubation 1 mL of DMSO was used to solubilize the formazan products. The optical density (OD) was measured at 570 nm in a microplate reader Epoch (BioTek). A percentage of viability was calculated as follows:

(5) $$\;\%\mathrm{Viability}=\frac{\left({\mathrm{OD}}_{570\mathrm{nm}}\mathrm{test}\;\mathrm{material}\right)\;-\;{\mathrm{OD}}_{570\mathrm{nm}}\mathrm{blank})}{\;\;\;\;\left({\mathrm{OD}}_{570\mathrm{nm}}\mathrm{control}\right)\;-\;{\mathrm{OD}}_{570\mathrm{nm}}\mathrm{blank})}$$

##### Con A/o-Pd Assay

Caco-2 cells were trypsinized, washed in saline solution and divided into three groups: (1) Control (2.5 × 10^5^ cells in 5 mL of saline solution); (2) BGT (2.5 × 10^5^ cells in 5 mL of BGT 12.5 mg mL^−1^); (3) PLOMW (2.5 × 10^5^ cells in 5 mL of PLMW 12.5 mg mL^−1^). Each group was incubated at 30 °C for 15 min, under gentle shaking. Then, the cells were washed twice with 5 mL Phosphate Buffered Saline (PBS) and centrifugated (2000 rpm) for 5 min. Subsequently, the cells were transferred to a clean tube and given a final wash. After an additional centrifugation (2000 rpm) for 5 min, the supernatant was removed. The pellet was stirred with a vortex mixer and washed twice with 12 mL of PBS and centrifugated (2000 rpm) for 5 min. Furthermore, the cells were transferred to a clean tube and washed prior to the addition of the next reagent (5 mL of PBS containing 1.0 mM calcium chloride and 10 mg L^−1^ biotinylated concanavalin A from Canavalia ensiformis (Con-A)). The mixture was incubated at 30 °C for 30 min under gentle shaking, centrifuged (2000 rpm) for 5 min. The cells were washed twice with PBS and transferred to a clean tube. 5 mL of PBS containing 5 mg/L streptavidin peroxidase was added and each tube was incubated at 30 °C for 60 min. Finally, the cells were washed and transferred to a clean tube and resuspended with 1 mL of o-phenylenediamine dihydrochloride (o-pd) solution (containing 0.4 mg o-pd and 0.4 μL 30% H_2_O_2_ in 1.0 mL 0.05 M citrate phosphate buffer). The oxidation of o-pd was stopped after 2 min with 1.0 mL of 1.0 M H_2_SO_4_ and the optical density measured at 490 nm (spectrophotometer Epoch, BioTek) [30].

### 2.6. Preparation of Puddings

Puddings were prepared using starch and PLOMW as gelling agent. Pears were purchased in a local supermarket and after washing, they were peeled. In order to prepare the puree, 50 mL of water was added to the fruit pulp (650 g) divided into smaller pieces. The pulp was then heated and stirred at low speed for 15 min, setting the temperature at 100 °C. Subsequently, after inactivation of the fruit enzymes, the heating was stopped and the pulp was ground for 1 min at high speed. The formulation of the pudding was determined after evaluating the consistency, according to the different quantity of gelling agent. Specifically, the pudding (PPLOMW) was prepared by mixing 100 mL of milk, 10 g of starch, 50 g of pear puree and 0.5 g of PLOMW. The starch was solubilized at room temperature in 75.0 mL of milk. The pear puree solubilized in the remaining volume of milk was then added under stirring at 80 °C for 5 min. The tare gum was gradually added without interrupting stirring and heating, to favor its hydration. The pudding was packaged in 60 g glass jars, immediately sealed with a metal lid. The pudding samples were stored at 4 °C in the refrigerator until the analysis carried out when the containers were opened (day 0), after seven, fourteen and twenty-eight days from the preparation. A control pudding (BCTG) was also prepared as control under the same conditions as described above, but using commercial tara gum (CTG) instead of PLOMW. 

### 2.7. Characterization of Pudding

#### 2.7.1. Rheological Characterization 

The rheometric measurements of the food matrices PPLOMW and PBTG were carried out using a strain-controlled RFS III rheometer (Rheometric Scientific Inc. at Piscataway, NJ, USA), equipped with plate-plate geometry: Gap 2 mm, Φ 25 mm. The temperature was controlled by means of a Peltier system (uncertainty of 0.1 °C). The samples were preliminarily subjected to strain sweep tests at a frequency of 1.0 Hz to determine the region of linear viscoelasticity (region in which the G′ and G″ vs strain modules are constant). The trend of the G′ and G″ vs. frequency (frequency sweep tests) in the linear viscoelasticity region was determined in the frequency range of 0.1–16.0 Hz.

#### 2.7.2. Antioxidant Performances of the Puddings

Pudding samples were analyzed, in terms of antioxidant properties, on the day they were prepared and after 7, 14 and 28 days, respectively [31]. Briefly, 10.0 g of pudding (PPLOMW or BCTG) were suspended in 20.0 mL of water by maceration for 24 h. The aqueous solution was then centrifuged at 8000 rpm for 10 min. Each extract was obtained by recovering the supernatant and was characterized in terms of APG, PAC and FC by the methodologies previously described, expressing the results as mg of CT per gram of pudding. Similarly, antioxidant performances were recorded by evaluation of TAC (expressed as mg of CT per gram of pudding) and scavenging activity of both aqueous (against ABTS radical) and organic (against DPPH radical) environments. Each measurement was performed in triplicate and data expressed as means (±SD).

### 2.8. Statistical Analysis

The inhibitory concentration 50 (IC_50_) was calculated by non-linear regression with the use of Prism Graph- Pad Prism, version 4.0 for Windows (GraphPad Software). One-way analysis of variance test (ANOVA) followed by a multicomparison Dunnett’s test were applied. All toxicity essays were performed in triplicate. Statistical analyses were made with Graph Pad. The data were evaluated by one-way analysis of variance followed by the Mann–Whitney U test. A value of *p* < 0.005 was considered significant.

## 3. Result and Discussion

### 3.1. Oil Mill Wastewater Treatment

Oil mill wastewater (OMW) displays a variable composition that is influenced by different issues including agronomic parameters, such as cultivar and maturation of the olive fruit, region of origin and climatic conditions [32]. In particular, OMW is an acidic and dark suspension mainly composed by water (83–94% *w*/*w*) and also containing inorganic (0.4–2.5% *w*/*w*) and organic substances (4–18 % *w*/*w*), including mucilage, lignin, tannins and pectins, as well as cation species such as magnesium, sodium, calcium and potassium. In addition, OMW contains phenolic compounds, the most popular high added-value ingredients, varying from 0.5 to 24 g/OMW), that represent about 98% (*w*/*w*) of the phenols typically present in olive fruit [33]. A high concentration of polyphenols generally involves a condensation step by ultrafiltration, thermal concentration or freeze-drying processes [34]. In this regard, OMWs from Roggianella *cv* were subjected to a freeze-drying process after preliminary filtration and centrifugation providing a vaporous solid (LOMW) that was deeply characterized by chromatographic and spectroscopic techniques, as well as in terms of antioxidant performance.

### 3.2. HPLC-MS/MS and ^1^H-NMR Analyses of Lyophilized Oil Mill Wastewater

Separation of the main polyphenols in LOMW was carried out by HPLC-MS/MS analysis and their identification was based on mass measurements of deprotonated [M–H]^−^ ions and MS/MS fragmentation patterns (Table 1). The compound at RT = 1.475 min (at concentration of 0.71 μg mL^−1^) was assigned to a verbascoside residue lacking the rhamnose moiety due to its [M–H]^−^ at *m*/*z* 477 together with the fragments at *m*/*z* 459 (water loss) and 161 (ascribable to the dehydrated ion of the caffeic acid unit), as already hypothesized in literature [31]. The compound at RT = 1.515 with a concentration of 0.19 μg mL^−1^, exhibiting [M–H]^−^ at *m*/*z* 169 and a fragment at *m*/*z* 151 probably produced by the loss [M–H–H_2_O]^−^, was tentatively identified as 3,4-dihydroxyphenylglycol [15]. 

Six phenolic acids and derivatives were also revealed, namely quinic acid (4.1 μg mL^−1^), HyEDA (0.27 μg mL^−1^) and decarboxymethyl-elenolic acid derivative (1.55 μg mL^−1^), hydroxylated product of dialdhydic form of decarboxymethyl elenolic acid (3.3 μg mL^−1^), caffeic acid (1.9 μg mL^−1^) and *p*-coumaric acid (2.1 μg mL^−1^) on the basis of their [M–H]^−^ ions as well as the presence of [M–H_2_O]^−^ and [M–CO–2H_2_O]^−^ in their fragmentation patterns [15,17]. The compounds at RT = 2.245 min and 2.470 min (having concentrations of 4.3 and 2.9 μg mL^−1^, respectively) were two 3-hydroxytyrosol glucoside isomers because they showed the same [M–H]^−^ at *m*/*z* 315 and similar MS/MS spectra characterized by two main fragment ions at *m*/*z* 153, which were formed through the loss of a glucose moiety and at *m*/*z* 123 corresponding to the subsequent loss of the CH_2_OH group. However, since different hydroxytyrosol glucoside isomers (i.e., hydroxytyrosol-1-O-glucoside, hydroxytyrosol-3′-O-glucoside and hydroxytyrosol-4′-O-glucoside) have been identified in *Olea europaea* [32], they were not furtherly distinguishable by MS analysis. Finally, oleuropein aglycone derivative (5.8 μg mL^−1^) and 3-hydroxytyrosol (0.09 μg mL^−1^) were recognized by matching chromatographic and MS characteristics to those of previous literature reports in the former case [15] and of a pure standard in the latter one.

**Figure 1:**
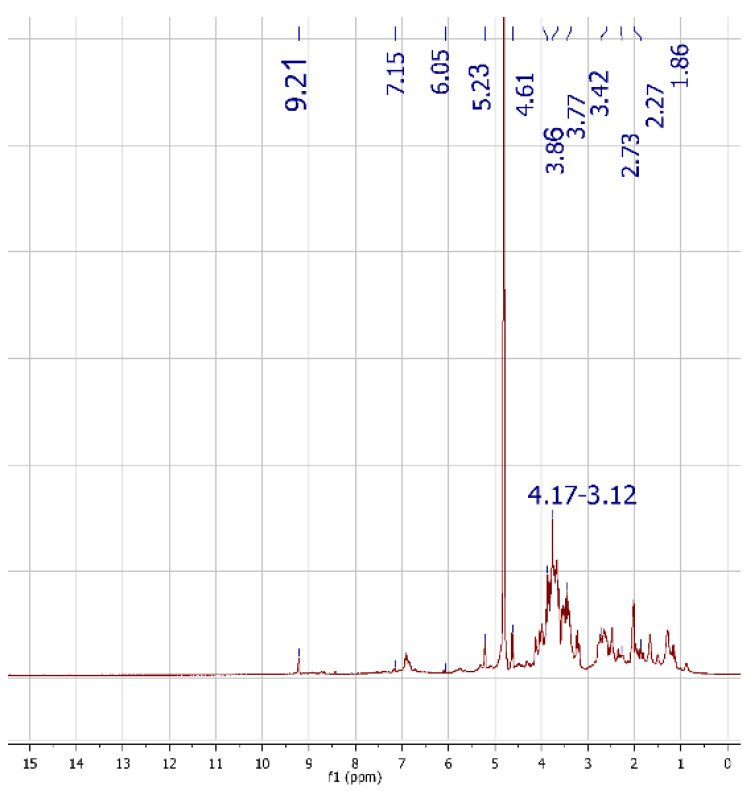
^1^H-NMR of 16.7 mg of LOMW sample in 0.6 μL of D_2_O.

As reported in Figure 1, the ^1^H-NMR of LOMW revealed the main features typical of minor components in olive oil reported in Table 2, responsible for its interesting biological activities. 

The corrected assignment was obtained comparing the NMR spectra with those available in literature and using the Human Metabolome Database (HMDB). 

Many signals belonging to sugar residues derived from glycosides and the OH group of Verbascoside, together with Ph-CH_2_ and CH_2_-O of Hydroxythyrosol and the CH_3_ and the enantiotopic CH_2_-OH of Oleuropein are detected. Furthermore, at 0.89, 1.3, 1.64, 2.02, 2.35 ppm, the signals of oleic acid are visible.

### 3.3. Antioxidant Properties of Lyophilized Oil Mill Wastewater

LOMW was characterized by evaluation of APG, FC, PAC and AC in order to provide a straight measure of the antioxidant potential of this by-product and the results are reported in Table 3. 

By-products from the olive oil extraction process are found to be particularly rich in phenolic compounds [35,36]. The available phenolic groups of LOMW was 75.0 mg CT per gram. This value appears in the same magnitude of other studies reported in literature, showing high concentration of phenolic compounds present in the OMWs [37,38]. Typically, during the extraction process, the highest (98%) amount of polyphenols in olive fruit can be found in the OMWs (0.5–24 g L^−1^ of OMW), while only 2% of them is in the oil phase [30]. A correlation of this parameter with literature data appears quite difficult because total polyphenol concentration is strictly related to many factors, such as type and region of origin, maturity of olives, method of extraction, climatic conditions and cultivation and processing techniques [39].

The analysis of FC in LOMW was carried out by using AlCl_3_ reagent and the results were expressed as milligrams of CT per gram of sample (Table 2). In plants, the number of glycoside fractions of these compounds can vary from one to three. In particular, flavonoids are found glycosylated with carbohydrates such as glucose or rhamnose, but they can also be found linked to glucose units such as galactose, arabinose or other sugars [40]. The results showed that phenolic compounds with a flavonoid structure in LOMW are equal to 34.0 mg CT per gram, corresponding to 45.3% of the APG.

The evaluation of PAC in LOMW was carried out using the Arnov’s method by expressing the results as milligrams of CT per gram of LOMW. Such compounds are hardly found in the free form, due to their ability to link quinic and tartaric acids forming esters and/or glycosylated derivatives [41]. LOMW sample provided high amounts of PAC (50.8 mg CT per gram), showing values that were equal to 67.7% of the total polyphenols. As can be seen from the data obtained by LC-MS analysis, the phenolic acid content was mainly related to the amounts of quinic, coumaric and caffeic acids. 

Finally, a colorimetric assay was employed to quantified anthocyanin compounds, a class natural pigments responsible for the coloring of most fruits and vegetables [42]. Recorded results displayed that AC in LOMW was equal to 0.15 mg CT g^−1^, almost two orders of magnitude lower than FC and PAC.

Antioxidant properties of the food matrix were deeply investigated by specific tests, including total antioxidant capacity and scavenging activity of the LOMW against DPPH and ABTS radicals. TAC of LOMW was determined using (NH_4_)_2_MoO_4_ reagent and by expressing the results as mg CT per gram of matrix. According to Prieto et al. (1999) [43], this reagent can be systematically applied for the evaluation, both in aqueous and organic environments, of the antioxidant activity of matrices with a complex composition.

The recorded results (Table 3) depicted as a high APG value was not always related to a significant antioxidant capacity according to literature data [44], highlighting as the class of phenolics deeply influenced the total antioxidant capacity of the food matrix. 

In order to determine scavenger activity both in aqueous and in organic environments, LOMW was tested against DPPH and ABTS radicals. The ability of LOMW to inhibit these reactive species, was expressed in terms of IC_50_ (mg mL^−1^), as reported in Table 3. In particular, the ability to inhibit the ABTS radical is a highly used parameter for the determination of antioxidant activity in food and biological samples [45]. Based on the inhibition kinetics of the ABTS radical, recorded LOMW IC_50_ value was 0.019 mg mL^−1^, almost five times less compared to the activity against DPPH, suggesting that LOMW is a matrix rich in highly hydrophilic moieties.

### 3.4. Synthesis of the Tara Gum Conjugate

An innovative strategy to synthesize versatile materials with antioxidant features involved the covalent linkage of the biomolecules of LOMW on the tara gum (TG) chain. Literature data largely proposed TG as starting materials in the synthesis of innovative polymeric carries of nutraceuticals able to be employed in the food industry [13,14,46], but to the best of our knowledge covalent grafting of bioactive molecules on TG chain was not investigated. This approach allowed the preparation of a high molecular weight antioxidant compounds showing improved chemical stability, as well as lower degradation rate compared to the low molecular weight antioxidants [47]. Conjugate polymers with antioxidant features were synthesized by employing an eco-compatible, radical initiated grafting procedure. Antioxidant conjugate was synthesized by anchoring the LOMW reactive species to TG chains, using a water soluble in a radical reaction initiated by a biocompatible redox pair (H_2_O_2_/ascorbic acid). This redox couple showed numerous advantages, such as the opportunity of inducing polymerization reaction at room temperature drastically reducing the risks of degradation of phenolic compounds and avoiding the generation of any type of toxic products [48]. A specific LOMW/TG ratio (*w*/*w*) was used in the polymerization mixture. In particular, a quantity of LOMW equivalent to 35 mg of catechin (calculated by APG value) for each gram of commercial TG was found to be optimal. After 24 h, to remove the unreacted molecules, the conjugate was subjected to a purification process by dialysis and the resulting solution was lyophilized which led to obtaining a vaporous solid (PLOMW) whose antioxidant properties were investigated. To verify the antioxidant performance of the conjugate, a control polymer (labelled BTG), was prepared in the same conditions, but in the absence of LOMW.

### 3.5. ^1^H-NMR Analysis of the Polymers

**Figure 2:**
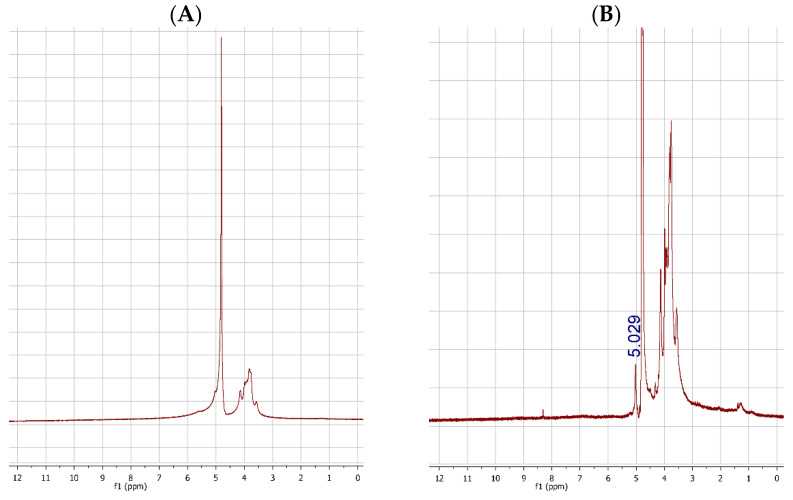
Panel (**A**): ^1^H-NMR spectrum of BTG; Panel (**B**): ^1^HNMR spectrum of PLOMW. In the panel (**B**), the signal at 5.029 ppm belongs to H_1_ of α-D-galactopyranose which in the panel (**A**) collapsed into the D_2_O signal.

The ^1^H-NMR spectra of BTG (Figure 2A) and PLOMW (Figure 2B) showed the main residues of galactomannan [49], scaffold of TG and some fragments of LOMW (close to 1.12–1.40 ppm and 8.32 ppm) that confirm the conjugation. 

### 3.6. Calorimetric Analysis of the Polymers

**Figure 3:**
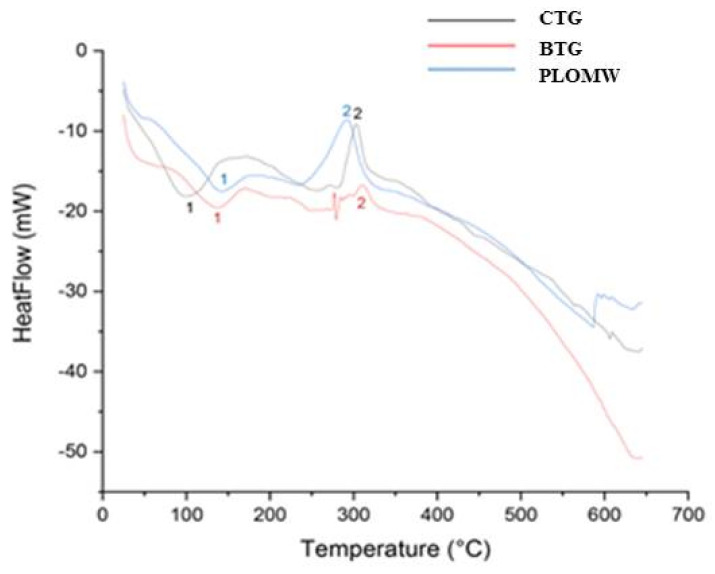
Differential scanning calorimetry (DSC) of PLOMW, BTG and CTG.

The Differential Scanning Calorimetry analysis of PLOMW, BTG and the commercial tara gum (CTG) was performed subjecting the samples to an isotherm at 25 °C for 20 min and heat ramp from 25 to 650 °C at a temperature scanning speed of 10 °C min^−1^ under nitrogen flow. The results obtained (Figure 3) allow to highlight the presence of two significant peaks in all the samples: an exothermic peak (100–150 °C) and an endothermic peak (around 300 °C). 

Specifically, the graph shows that the two polymer samples show the same peaks as TG sample, but the values are slightly shifted with respect to the reference (CTG) and this phenomenon is most likely due to the different molecular structure.

The enthalpy was then calculated for each peak and the results were reported in Table 4. 

Measuring the enthalpy of fusion allows to calculate the degree of crystallinity of a substance. Therefore, a higher enthalpy corresponds to a greater interaction between the molecules. The results show that as regards the enthalpy values measured on the peak (1), these are significantly lower for the PLOMW and BTG polymers. This behavior denotes a lower level of interaction between the polymer molecules than the commercial tare rubber sample. On the other hand, the values recorded for the peak (2) show how the blank polymer has a higher enthalpy value (−11.6 J g^−1^) and, therefore, has a greater degree of interaction between its molecules than CTG and PLOMW polymer. In particular, the latter, having the lowest enthalpy value (−154.3 J g^−1^), is the one with the least interaction between its molecules.

### 3.7. Toxicity Evaluation of the Conjugate

#### 3.7.1. NRU Assay

**Figure 4:**
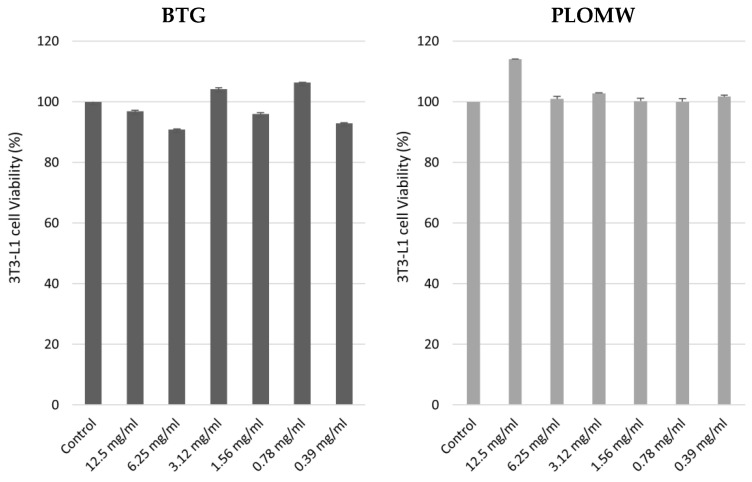
3T3 cells viability (%) measured through NRU cytotoxicity assay upon treatment with increased concentrations of BGT and PLOMW (mg/mL). Each column represents the mean + SD of 3 wells/group.

The effect of BGT and PLOMW on 3T3 fibroblasts was measured by *in vitro* acute toxicity assay through the neutral red uptake (NRU, Figure 4). The treatment with increased concentrations of BGT and PLOMW non-altered cell viability compared to the control, in both treatments. 

#### 3.7.2. Cell Viability by MTT Assay

To evaluate the cytotoxic and adverse cellular effects of BGT and PLOMW, was used the MTT assay. This test assesses the activity of mitochondrial enzymes in healthy cells by measuring the absorbance of purple formazan, formed through an NADP-dependent reaction catalyzed by succinate dehydrogenase in metabolically active cells [50].

**Figure 5:**
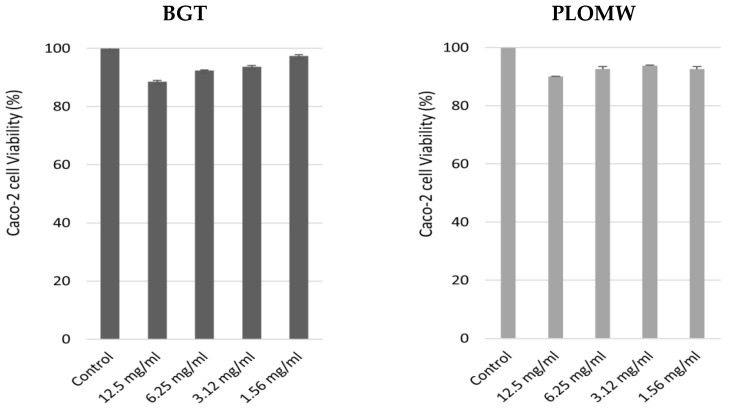
Effect of BTG and PLOMW on Caco-2 cells viability.

As shown in Figure 5, the treatments with increased concentrations of BTG and PLOMW for 24 h, not altered Caco-2 cells viability.

#### 3.7.3. Bio-Adhesive Ability Assay

**Figure 6:**
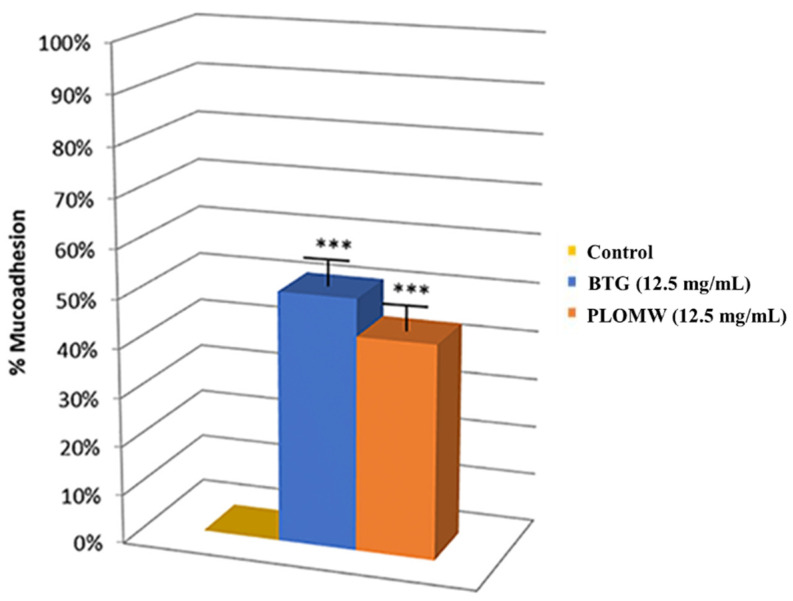
Muco-adhesion of BTG and PLOMW determined as reduction (%) of lectin binding on Caco-2 cells at different concentrations; *** *p* < 0.001 vs. control.

In this study, to evaluate the bio-adhesive ability of active substances was used a technique described by Rizza [30]. Our results showed a similar muco-adhesivity of BTG and PLOMW (Figure 6) at 12.5 mg mL^−1^ concentration. This concentration represents the final dose of dietary use.

### 3.8. Antioxidant Performances

Antioxidant properties of PLOMW were explored by confirming that the grafting reaction has taken place and also to verify whether the reaction conditions were harmful to the active compounds in LOMW. Total phenolic groups, available phenolic acids and antioxidant activity of the conjugate have been reported in Table 5.

Data confirmed that the grafting reaction avoids the loss of the antioxidant activity of LOMW, while BTG did not demonstrate any interference. Specifically, the analyses of the inhibition kinetics against radical species underlined the good performance of PLOMW, both in organic and aqueous environments. However, the response towards the radical ABTS appears almost three times higher, as confirmed by the IC_50_ values. The collected data clearly indicates as PLOMW is able to guarantee good results and that when introduced into a food, it can impart significant biological properties from a nutritional point of view, producing beneficial effects on the health of consumers.

### 3.9. Pudding Preparation and Evaluation of the Antioxidant Properties

Gelling properties of the conjugate suggest the employment of this macromolecular system for the preparation of suitable functional foods with high added value. In particular, the PLOMW was used for the production of a fruit-based pudding. The fruit gives numerous benefits to the human body often related to the presence of phenolic compounds. Different studies have correlated the high intake of fruit with the lower incidence of cardiovascular, neurodegenerative and chronic diseases, such as cancer and diabetes [51]. To highlight the effect and benefits derived from using PLOMW in the puddinG′s preparation, the pear, not particularly rich in antioxidant molecules, was chosen as raw material [52]. Although it can contain in the range 27–41 mg of phenols per 100 g of pulp, the antioxidant profile of pear remains distant from other fruits such as blackberry, raspberry, blueberry, strawberry, cherry and grape [53]. At the same time, this fruit is very digestible and tasty, especially in the preparation of fruit juices for children or hospitalized patients. In this context, puddings based on pear puree were prepared using PLOMW and CTG as gelling agent and their antioxidant capacity was investigated as a function of time. For this purpose, each pudding jar was opened at set time intervals and subjected to an extraction process according to a procedure reported in literature, with some modifications [31]. Antioxidant properties of PPLOMW were analyzed at the opening day (t = 0), after 7, 14 and 28 days. The data of total phenolic groups, phenolic acids and the scavenging properties in aqueous environment are reported in Table 6.

Available phenolic groups (t = 0 days), expressed as mg of CT per gram of pudding, confirm that the addition of the antioxidant polymer PLOMW as gelling agent, significantly increases (almost two times) APG value of PCTG. Similarly, PPOMW at time zero displayed an increased amount of available phenolic acids and more performing scavenging capacity against the hydrophilic radical ABTS. The analysis of the trend of these parameters over 28 days, in terms of APG, highlighted the slight decrease observed for PPOMW (equal to 11% after 28 days). On the contrary, APG decrease equal to 30% after 28 days was recorded with the pudding prepared using CTG. This finding was mainly interesting because it highlights that the employment of the conjugate increased phenolic groups in the functional food and maintained their concentration over time. The same trend was recorded in the content of available phenolic acids, with a decrease after 28 days of 76% for PPLOMW matrix, which increased to 82% in PCTG.

**Figure 7:**
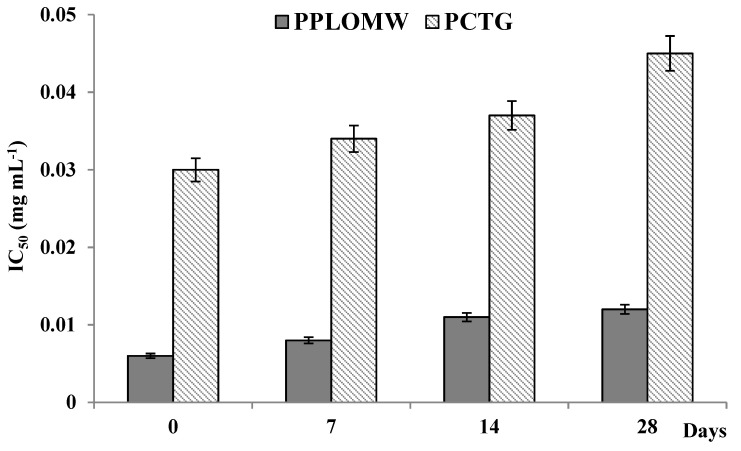
IC_50_ trend as function of the time of PPLOMW and PCTG.

Finally, by recording the inhibition profiles towards the ABTS of the puddings over time, it was observed that the enriched pudding proved to be more effective against this radical specie, compared to the control (Figure 7). IC_50_ values recorded for the PPLOMW underwent a slight decrease over time (0.0124 mg mL^−1^, after 28 days). On the contrary, the IC_50_ values recorded for the pudding based on CTG, underwent a greater decrease (Figure 7).

Ultimately, the polymeric conjugate, synthesized using TG and compounds with a polyphenolic structure obtained from waste products of the oil extraction process, inserted in a food matrix such as pudding with pear, has guaranteed it a greater antioxidant capacity and the possibility of keeping it almost unchanged over time.

### 3.10. Rheological Analysis of Puddings

Rheology is the science that studies the deformation and flow of matter in liquid or solid form, i.e., the response of materials to mechanical stress in terms of viscosity and elasticity [54]. The rheometric measurements of the food matrices PPLOMW and PCTG were carried out by recording the frequency sweeps of the G′ and G″ modules as a function of the frequency in the linear viscoelasticity region. The trend of G′ and G″ vs. the frequency is also called the mechanical spectrum and allows a quantitative rheological characterization of the materials. 

Such a linear viscoelastic behavior has been previously observed in gel systems. All mixtures show a typical gel-like response in which G′ is higher than G′’. Higher values of G′ may reflect the stronger interactions existing among the domains which favor the formation of highly elastic lattices. In order to obtain quantitative information, the weak gel model was applied, which models the system as consisting of connected rheological units. This allowed to determine the values of “A” and “z”, where “A” represents the interaction force between the rheological units and “z” the coordination number, that is, the number of rheological units interacting with a reference unit. The “A” and “z” values of the PPLOMW and PCGT food matrices determined by frequency sweep test are shown in Table 7.

As can be seen from the data reported in Table 7, the values of “A” recorded for PCTG, both at 5 and 25 °C increase as functions of time, while the values of “z” are very similar in the first fourteen days and then significantly increased after twenty-eight days. This indicates that initially, the network formed increases the strength between the links and then expands three-dimensionally over time. 

**Figure 8:**
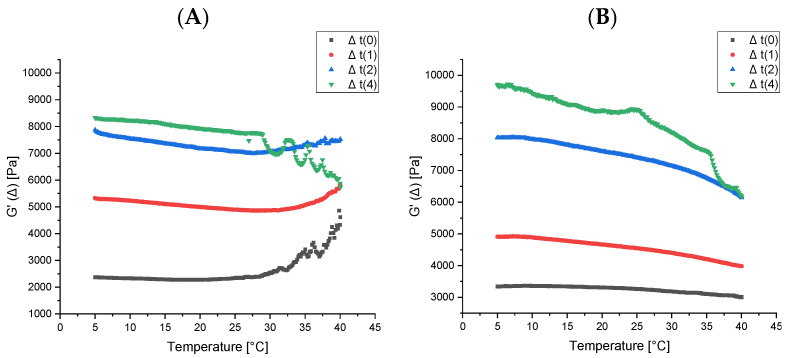
Time Cure test for the PCGT (**A**) and PPLOMW (**B**) food matrix over time (weeks).

Finally, for each sample, temperature ramp tests or time cures were carried out to analyze the behavior of the system as a function of temperature and the results are shown in Figure 8A,B.

In this type of test, G′ and G″ are recorded as a function of temperature at a frequency of 1 Hz within the linear viscoelastic region. The temperature range investigated was 5–40 °C, with a scanning speed of 1 °C min^−1^. Figure 8 shows the G′ comparison (elastic modulus) of the pudding samples prepared with the two polymers investigated (PPLOMW and PCGT) over time. The recorded data indicate that at “time zero” the samples have very different values of elastic modulus (G′). In fact, the G′ values recorded for the PCGT sample are lower than those observed for PPLOMW. However, with the passage of time, the difference between the modules tends to decrease and overlap when both samples reach two weeks of maturation. It is important to note that as the maturation time increases (beyond two weeks), the elastic values of the PPLOMW sample are greater. In fact, the pudding prepared with PPLOMW at “time 4 weeks” shows the highest elastic modulus. This rheological behavior can be attributed to a greater structuring effect induced by the conjugated polymer. Preliminary data suggest that water may play a central role in the structuring through hydrogen bonds, a hypothesis that must necessarily be proved by means of spectroscopic techniques.

## 4. Conclusions

Olive mill wastewaters (OMW) were explored as inexpensive, precious and valuable sources of bioactive molecules to be employed in the production of antioxidant-enriched milk-based products. In this regard, lyophilized OMW (LOMW) were involved in a radical grafting reaction to synthesize a tara gum conjugate, suitable as a thickening agent in the preparation of pear puree-based pudding. Chemical composition of LOMW was evaluated by ^1^H-NMR and HPLC-MS/MS analyses and oleuropein aglycone derivative (5.8 μg mL^−1^) was detected as the main compound. Additionally, this olive process by-product was characterized by evaluation of the total phenolic content, flavonoids, phenolic acids and anthocyanins amount, providing a straight measure of its antioxidant features. LOMW was able to inhibit ABTS radical, displaying in the aqueous medium, scavenger properties almost one order of magnitude increased compared to the organic one. LOMW reactive species and tara gum chains were involved in an eco-friendly grafting reaction to synthesize a polymeric conjugate that was characterized by spectroscopic, calorimetric and toxicity studies. Antioxidant properties of the polymeric conjugate were also evaluated, suggesting its employing as a thickening agent in the preparation of pear puree-based pudding. Milk-based foodstuff showed high performance of consistency and relevant antioxidant features over time (28 days) in comparison with its non-functional counterparts confirming LOWM as an attractive source for the development of functional foods.

## Figures and Tables

**Table 1:** Identified polyphenol compound in LOMW (in μg mL^−1^). Data represent mean ± RSD (*n* = 3).

Compound	RT (min)	LOMW (μg mL^−1^)
Verbascoside residue	1.475	0.71 ± 0.06
3,4-dihydroxyphenylglycol	1.515	0.185 ± 0.017
Quinic acid	1.773	4.1±0.4
3-hydroxytyrosol glucoside isomers 1	2.245	4.3 ± 0.4
3-hydroxytyrosol glucoside isomers 2	2.470	2.9 ± 0.3
Dimer 407	2.610	1.56 ± 0.14
3-hydroxytyrosol	2.957	0.092 ± 0.008
Decarboxymethyl-elenolic acid derivative	3.542	1.55 ± 0.14
Hydroxylated product of dialdhydic form of decarboxymethyl elenolic acid	4.428	3.3 ± 0.3
Caffeic acid	7.996	1.91 ± 0.17
Decarboxymethyl-elenolic acid (HyEDA)	8.075	0.27 ± 0.02
Oleuropein aglycone derivative	8.124	5.8 ± 0.5
*p*-coumaric acid	10.798	2.13 ± 0.19

RT = retention time; LOMW = Lyophilized olive mill wastewater.

**Table 2:** Characterization of LOMW by ^1^H-NMR spectroscopy.

Compound	Assignment	Chemical Shift (ppm)
Verbascoside	OH-18; OH-17	s 9.21; 4.61
Verbascoside	O-CH-O	d 5.23
Sugar residue of glycosides	CH-OH	m 4.17–3.12
Hydroxythyrosol	Ph-CH_2_-	2.73
Hydroxythyrosol	CH_2_-O	3.76
Oleuropein	CH_3_	s 3.86
Oleuropein	Enantiotopic CH_2_-OH	m 3.42
Caffeic acid	CH_2_=CH_2_	dd 6.05–7.15
Quinic acid	Enantiotopic CH_2_-CH	2.27–1.86

**Table 3:** Antioxidant characterization of LOMW. Data represent mean ± RSD (*n* = 3).

Sample	APG (mg CT/g)	PAC (mg CT/g)	FC (mg CT/g)	AC (mg CT/g)	TAC (mg CT/g)	IC_50_ (mg mL^−1^)	
						DPPH Radical	ABTS Radical
LOMW	75.0 ± 0.7	50.8 ± 0.4	34.0 ± 0.4	0.15 ± 0.01	1.10 ± 0.05	0.095 ± 0.003	0.019 ± 0.001

LOMW = Lyophilized olive mill wastewater; APG = Available phenolic groups; PAC = Phenolic acids content; FC = Flavonoid content; AC = Anthocyanin content; TAC = Total antioxidant activity; DPPH = 2,2-diphenyl-1-picrylhydrazyl radical; ABTS = 2,2′-azino-bis(3-ethylbenzothiazolin-6-sulphonic radical; CT = catechin.

**Table 4:** Enthalpy and temperature values of the polymers and commercial tare rubber peaks.

Sample	T Center Peak 1 (°C)	T Center Peak 2 (°C)	Enthalpy Peak 1 (J g^−1^)	Enthalpy Peak 2 (J g^−1^)
PLOMW	141.0	311.8	125.4	−154.3
BTG	137.7	291.4	76.0	−11.6
CTG	100.4	302.6	261.4	−83.4

PLOMW = Polymer conjugate lyophilized olive mill wastewater and tara gum; BTG = Blank tara gum; CTG = commercial tara gum.

**Table 5:** Total polyphenols, phenolic acid contents and antioxidant activity of the conjugate polymer. Data represent mean ± RSD (*n* = 3).

Sample	APG (mg CT/g)	PAC (mg CT/g)	TAC (mg CT/g)	IC_50_ (mg mL^−1^)	
				DPPH Radical	ABTS Radical
PLOMW	16.3 ± 0.5	15.7 ± 0.4	2.26 ± 0.11	0.322 ± 0.005	0.106 ± 0.005
BTG	-	-	-	-	-

PLOMW = Tara gum grafted with lyophilized olive mill wastewater; APG = Available phenolic groups; PAC = Phenolic acids content; TAC = Total antioxidant activity; DPPH = 2,2-diphenyl-1-picrylhydrazyl radical; ABTS = 2,2′-azino-bis(3-ethylbenzothiazolin-6-sulphonic radical; CT = catechin.

**Table 6:** Total polyphenols, phenolic acid contents and antioxidant activity of puddings based on PLOMW and CTG. Data represent mean ± RSD (*n* = 3).

Time (Days)	Pudding	APG (mg CT/g Pudding)	PAC (mg CT/g Budino)	IC_50_ (mg mL^−1^)
				ABTS Radical
0	PPLOMW	0.220 ± 0.009 ^a^	0.261 ± 0.011 ^a^	0.0060 ± 0.0003 ^a^
	PCTG	0.113 ± 0.005 ^b^	0.244 ± 0.009 ^b^	0.0324 ± 0.0011 ^b^
7	PPLOMW	0.193 ± 0.007 ^c^	0.136 ± 0.005 ^c^	0.0082 ± 0.0003 ^c^
	PCTG	0.101 ± 0.004 ^d^	0.070 ± 0.002 ^d^	0.0345 ± 0.0015 ^b^
14	PPLOMW	0.194 ± 0.006 ^c^	0.075 ± 0.002 ^e^	0.0118 ± 0.0005 ^d^
	PCTG	0.089 ± 0.003 ^d^	0.056 ± 0.002 ^f^	0.0397 ± 0.0018 ^e^
28	PPLOMW	0.196 ± 0.006 ^c^	0.062 ± 0.002 ^g^	0.0124 ± 0.0006 ^d^
	PCTG	0.080 ± 0.002 ^e^	0.042 ± 0.001 ^h^	0.0453 ± 0.0021 ^f^

PPLOMW = Pudding based on tara gum grafted with lyophilized olive mill wastewater; PCTG = Pudding based on commercial tara gum; APG = Available phenolic groups; PAC = Phenolic acids content; ABTS = 2,2′-azino-bis(3-ethylbenzothiazolin-6-sulphonic radical; CT = catechin. Different letters express significant differences (*p* < 0.05).

**Table 7:** Frequency sweep test for PPLOMW food matrices and PCGT over time.

Sample	A ± 100	z ± 1
5 °C PCGT (t = 28 days)	8800	18
5 °C PCGT (t = 14 days)	8200	12
5 °C PCGT (t = 7 days)	5600	11
5 °C PCGT (t = 0 days)	2800	11
25 °C PCGT (t = 28 days)	7200	45
25 °C PCGT (t = 14 days)	7300	12
25 °C PCGT (t = 7 days)	4800	11
25 °C PCGT (t = 0 days)	2000	11
5 °C PPLOMW (t = 28 days)	9600	18
5 °C PPLOMW (t = 14 days)	8900	10
5 °C PPLOMW (t = 7 days)	5100	11
5 °C PPLOMW (t = 0 days)	3400	14
25 °C PPLOMW (t = 28 days)	8200	32
25 °C PPLOMW (t = 14 days)	7200	12
25 °C PPLOMW (t = 7 days)	4400	12
25 °C PPLOMW (t = 0 days)	1100	7

PPLOMW = Pudding by polymer conjugate; PCTG = pudding by commercial tara gum.

## Data Availability

Not applicable.

## Supplementary Materials

The following supporting information can be downloaded at: www.mdpi.com/article/10.3390/foods11020158/s1, Table S1: Acquisition parameters for MRM HPLC-MS/MS analyses.

## References

[1] Spizzirri U.G., Aiello F., Carullo G., Facente A., Restuccia D. (2021). Nanotechnologies: An Innovative Tool to Release Natural Extracts with Antimicrobial Properties. Pharmaceutics.

[2] Roselli L., Cicia G., Cavallo C., Del Giudice T., Carlucci D., Clodoveo M.L., De Gennaro B.C. (2018). Consumers’ willingness to buy innovative traditional food products: The case of extra-virgin olive oil extracted by ultrasound. Food Res. Int..

[3] Mallamaci R., Budriesi R., Clodoveo M.L., Biotti G., Micucci M., Ragusa A., Curci F., Muraglia M., Corbo F., Franchini C. (2021). Olive Tree in Circular Economy as a Source of Secondary Metabolites Active for Human and Animal Health Beyond Oxidative Stress and Inflammation. Molecules.

[4] De Luca M., Restuccia D., Clodoveo M.L., Puoci F., Ragno G. (2016). Chemometric analysis for discrimination of extra virgin olive oils from whole and stoned olive pastes. Food Chem..

[5] Amirante P., Clodoveo M.L., Tamborrino A., Leone A., Paice A.G. (2010). Influence of the crushing system: Phenol content in virgin olive oil produced from whole and de-stoned pastes. Olives and Olive Oil in Health and Disease Prevention.

[6] Cirillo G., Curcio M., Spizzirri U.G., Vittorio O., Valli E., Farfalla A., Leggio A., Nicoletta F.P., Iemma F. (2019). Chitosan-Quercetin Bioconjugate as Multi-Functional Component of Antioxidants and Dual-Responsive Hydrogel Networks. Macromol. Mater. Eng..

[7] Protte K., Weiss J., Hinrichs J., Knaapila A. (2019). Thermally stabilised whey protein-pectin complexes modulate thethermodynamic incompatibility in hydrocolloid matrixes: A feasibility-study on sensory and rheological characteristics in dairy desserts. LWT-Food Sci. Technol..

[8] Lim H.S., Narsimhan G. (2006). Pasting and rheological behavior of soy protein-based pudding. LWT-Food Sci. Technol..

[9] Alamprese C., Mariotti M. (2011). Effects of different milk substitutes on pasting, rheological and textural properties of puddings. LWT-Food Sci. Technol..

[10] Ares G., Baixauli R., Sanz T., Varela P. (2009). New functional fibre in milk puddings: Effect on sensory properties and consumers’ acceptability. LWT-Food Sci. Technol..

[11] Wu Y., Ding W., He Q. (2018). The gelation properties of tara gum blended with κ-carrageenan or xanthan. Food Hydrocoll..

[12] Huamaní-Meléndez V.J., Mauro M.A., Darros-Barbosa R. (2021). Physicochemical and rheological properties of aqueous Tara gum solutions. Food Hydrocoll..

[13] Menegon Rosário F., Biduski B., Fernando dos Santos D., Hadlish E.V., Tormen L., Fidelis dos Santos G.H., Zanella Pinto V. (2021). Red araçá pulp microencapsulation by hydrolyzed pinhão starch, and tara and arabic gums. J. Sci. Food Agric..

[14] Ma Q., Ren Y., Wang L. (2017). Investigation of antioxidant activity and release kinetics of curcumin from tara gum/polyvinyl alcohol active film. Food Hydrocoll..

[15] D’Antuono I., Kontogianni V.G., Kotsiou K., Linsalata V., Logrieco A.F., Tasioula-Margari M., Cardinali A. (2014). Polyphenolic characterization of olive mill wastewaters, coming from Italian and Greek olive cultivars, after membrane technology. Food Res. Int..

[16] Leouifoudi I., Harnafi H., Zyad A. (2015). Olive mill waste extracts: Polyphenols content, antioxidant, and antimicrobial activities. Adv. Pharmacol. Sci..

[17] De Santis S., Liso M., Verna G., Curci F., Milani G., Faienza M.F., Franchini C., Moschetta A., Chieppa M., Clodoveo M.L. (2021). Extra Virgin Olive Oil Extracts Modulate the Inflammatory Ability of Murine Dendritic Cells Based on Their Polyphenols Pattern: Correlation between Chemical Composition and Biological Function. Antioxidants.

[18] Carullo G., Scarpelli F., Belsito E.L., Caputo P., Rossi Oliverio C., Mincione A., Leggio A., Crispini A., Restuccia D., Spizzirri U.G. (2020). Formulation of New Baking (+)-Catechin Based Leavening Agents: Effects on Rheology, Sensory and Antioxidant Features during Muffin Preparation. Foods.

[19] Gawlik-Dziki U., Dziki D., Baraniak B., Lin R. (2009). The Effect of Simulated Digestion In Vitro on Bioactivity of Wheat Bread with Tartary Buckwheat Flavones Addition. LWT-Food Sci. Technol..

[20] Ardestani A., Yazdanparast R. (2007). Antioxidant and free radical scavenging potential of Achillea santolina extracts. Food Chem..

[21] Rababah T.M., Al-Omoush M., Brewer S., Alhamad M., Yang W., Alrababah M., Al-Ghzawi A.A.-M., Al-U’datt M., Ereifej K., Alsheyab F. (2014). Total phenol, antioxidant activity, flavonoids, anthocyanins and color of honey as affected by floral origin found in the arid and semiarid mediterranean areas. J. Food Process. Preserv..

[22] Giusti M.M., Wrolstad R.E., Wrolstad R.E. (2001). Anthocyanins: Characterization and measurement with W-visible spectroscopy. Current Protocols in Food Analytical Chemistry.

[23] Spizzirri U.G., Carullo G., Aiello F., Paolino D., Restuccia D. (2021). Valorisation of olive oil pomace extracts for a functional pear beverage formulation. Int. J. Food Sci. Technol..

[24] Spizzirri U.G., Carullo G., De Cicco L., Crispini A., Scarpelli F., Restuccia D., Aiello F. (2019). Synthesis and characterization of a (+)-catechin and L-(+)-ascorbic acid cocrystal as a new functional ingredient for tea drinks. Heliyon.

[25] Kellett M.E., Greenspan P., Pegg R.B. (2018). Modification of the cellular antioxidant activity (CAA) assay to study phenolic antioxidants in a Caco-2 cell line. Food Chem..

[26] Restuccia D., Giorgi G., Spizzirri U.G., Sciubba F., Capuani G., Rago V., Carullo G., Aiello F. (2019). Autochthonous white grape pomaces as bioactive source for functional jams. Int. J. Food Sci. Technol..

[27] Spizzirri U.G., Altimari I., Puoci F., Parisi O.I., Iemma F., Picci N. (2011). Innovative antioxidant thermo-responsive hydrogels by radical grafting of catechin on inulin chain. Carbohydr. Polym..

[28] Stokes W.S., Casati S., Strickland J., Paris M. (2008). Neutral Red Uptake Cytotoxicity Tests for Estimating Starting Doses for Acute Oral Toxicity Tests. Curr. Protoc. Toxicol..

[29] Mosmann T. (1983). Rapid colorimetric assay for cellular growth and survival: Application to proliferation and cytotoxicity assays. J. Immunol. Methods.

[30] Rizza L., Frasca G., Nicholls M., Puglia C., Cardile V. (2012). Caco-2 cell line as a model to evaluate mucoprotective proprieties. Int. J. Pharm..

[31] Sun Y., Hayakawa S., Ogawa M., Izumori K. (2007). Antioxidant properties of custard pudding dessert containing rare hexose, D-psicose. Food Control.

[32] Paraskeva P., Diamadopoulos E. (2006). Technologies for olive mill wastewater (OMW) treatment: A review. J. Chem. Technol. Biotechnol..

[33] He J., Alister-Briggs M., Lyster T.D., Jones G.P. (2012). Stability and antioxidant potential of purified olive mill wastewater extracts. Food Chem..

[34] Rahmanian N., Jafari S.M., Galanakis C.M. (2013). Recovery and Removal of Phenolic Compounds from Olive Mill Wastewater. J. Am. Oil Chem. Soc..

[35] Cardinali A., Pati S., Minervini F., D’Antuono I., Linsalata V., Lattanzio V. (2012). Verbascoside, isoverbascoside, and their derivatives recovered from olive mill wastewater as possible food antioxidants. J. Agric. Food Chem..

[36] Obied H.K., Bedgood D.R., Prenzler P.D., Robards K. (2007). Chemical screening of olive biophenol extracts by hyphenated liquid chromatography. Anal. Chim. Acta.

[37] Kiritsakis K., Rodríguez-Pérez C., Gerasopoulos D., Segura- Carretero A. (2017). Olive oil enrichment in phenolic compounds during malaxation in the presence of olive leaves or olive mill wastewater extracts. Eur. J. Lip. Sci. Technol..

[38] Mattonai M., Vinci A., Degano I., Ribechini E., Franceschi M., Modugno F. (2019). Olive mill wastewaters: Quantitation of the phenolic content and profiling of elenolic acid derivatives using HPLC-DAD and HPLC/MS^2^ with an embedded polar group stationary phase. Nat. Prod. Res..

[39] Ghanbari R., Anwar F., Alkharfy K.M., Gilani A.H., Saari N. (2012). Valuable Nutrients and Functional Bioactives in Different Parts of Olive (*Olea europaea* L.). Int. J. Mol. Sci..

[40] Andrés-Lacueva C., Medina-Remon A., Llorach H., Urpi-Sarda R., Khan M., Chiva-Blanch N., Zamora-Ros G., Rotches-Ribalta R., Lamuela-Raventòs R.M., De la Rosa L., Alvarez-Parrilla E., Gonzalez-Aguilar G.A. (2010). Phenolic Compounds: Chemistry and Occurrence in Fruits and Vegetables. Fruit and Vegetable Phytochemicals: Chemistry, Nutritional Value and Stability.

[41] Manach C., Scalbert A., Morand C., Rémésy C., Jiménez L. (2004). Polyphenols: Food sources and bioavailability. Am. J. Clin. Nutr..

[42] De Pascual-Teresa S., Moreno D.A., Garcìa-Viguera C. (2010). Flavanols and Anthocyanins in Cardiovascular Health: A Review of Current Evidence. Int. J. Mol. Sci..

[43] Prieto P., Pineda M., Aguilar M. (1999). Spectrophotometric quantitation of antioxidant capacity through the formation of a phosphomolybdenum complex: Specific application to the determination of vitamin E. Anal. Biochem..

[44] Giovanelli G., Brenna O.V. (2007). Evolution of some phenolic components, carotenoids and chlorophylls during ripening of three Italian grape varieties. Eur. Food Res. Technol..

[45] Floegel A., Kim D.O., Chung S.J., Koo S.I., Chun O.K. (2011). Comparison of ABTS/DPPH assays to measure antioxidant capacity in popular antioxidant-rich US foods. J. Food Compost. Anal..

[46] Rutz J.K., Zambiazi R.C., Borges C.D., Krumreich F.D., Da Luz S.R., Hartwig N., Da Rosa C.G. (2013). Microencapsulation of purple Brazilian cherry juice in xanthan, tara gums and xanthan-tara hydrogel matrixes. Carbohydr. Polym..

[47] Kurisawa M., Chung J.E., Uyama H., Kobayashi S. (2003). Enzymatic synthesis and antioxidant properties of poly(rutin). Biomacromolecules.

[48] Toti U.S., Aminabhavi T.M. (2004). Synthesis and characterization of polyacrylamidegrafted sodium alginate membranes for pervaporation separation of water + isopropanol mixtures. J. Appl. Polym. Sci..

[49] Chouana T., Pierre G., Vial C., Gardarin C., Wadouachi A., Cailleu D., Le Cerf D., Boual Z., Ould El Hadj M.D., Michaud P. (2017). Structural characterization and rheological properties of a galactomannan from Astragalus gombo Bunge seeds harvested in Algerian Sahara. Carbohydr. Polym..

[50] Liu Y., Nair M.G. (2010). An efficient and economical MTT assay for determining the antioxidant activity of plant natural product extracts and pure compounds. J. Nat. Prod..

[51] Salta J., Martins A., Santos R.G., Neng N.R., Nogueira J.M.F., Justino J., Rauter A.P. (2010). Phenolic composition and antioxidant activity of Rocha pear and other pear cultivars—A comparative study. J. Funct. Foods.

[52] Reiland H., Slavin J. (2015). Systematic Review of Pears and Health. Nutr. Today.

[53] Marinova D., Ribarova F., Atanassova M. (2005). Total phenolics and total flavonoids in bulgarian fruits and vegetables. J. Univ. Chem. Technol. Metall..

[54] Barnes H.A., Hutton J.F., Walters K. (1989). An Introduction to Rheology.

