# Supplementary material for: A Tara Gum/Olive Mill Wastewaters Phytochemicals Conjugate as a New Ingredient for the Formulation of an Antioxidant-Enriched Pudding
Source: Foods. 2022 Jan 8;11(2):158. doi: 10.3390/foods11020158 (PMC8774902; doi:10.3390/foods11020158)
Supplement: Supplementary file 1 [file foods-11-00158-s001.zip › foods-1479352-supplementary.pdf]

SUPPORTING INFORMATIONS

Table S1. Acquisition parameters for MRM HPLC-MS/MS analyses.

Compound	RT (min)	Q_1 (*m/z*)	Q_3 (*m/z*)	Frag (V)	CE (V)
Verbascoside residue	1.475	477	459	100	15
3,4-dihydroxyphenylglycol	1.515	169	123	100	15
Quinic acid	1.773	191	85	100	30
3-hydroxytyrosol glucoside isomer 1	2.245	315	153	80	15
3-hydroxytyrosol glucoside isomer 2	2.470	315	153	80	15
3-hydroxytyrosol	2.957	153	123	100	15
Decarboxymethyl-elenolic acid derivative	3.542	185	111	100	15
Hydroxylated product of dialdhydic form of decarboxymethyl elenolic acid	4.428	199	111	100	15
Caffeic acid	7.996	179	135	100	15
Decarboxymethyl-elenolic acid (HyEDA)	8.075	183	139	100	15
Oleuropein aglycone derivative	8.124	377	197	80	15
Coumaric acid	10.798	163	119	100	15

RT, retention time; Q_1, parent ion mass; Q_3, daugther ion mass; Frag, fragmentor voltage; CE, collision energy.
